# Epidemiology of Advance Directives in Extended Care Facility Patients Presenting to the Emergency Department

**DOI:** 10.5811/westjem.2015.8.25657

**Published:** 2015-11-16

**Authors:** Jessica Wall, Brian Hiestand, Jeffrey Caterino

**Affiliations:** * Penn Presbyterian Medical Center, Department of Emergency Medicine, Philadelphia, Pennsylvania; † Wake Forest School of Medicine, Department of Emergency Medicine, Winston-Salem, North Carolina; ‡ Ohio State University, Department of Emergency Medicine, Columbus, Ohio

## Abstract

**Introduction:**

We conducted an epidemiologic evaluation of advance directives and do-not-resuscitate (DNR) prevalence among residents of extended care facilities (ECF) presenting to the emergency department (ED).

**Methods:**

We performed a retrospective medical record review on ED patients originating from an ECF. Data were collected on age, sex, race, triage acuity, ED disposition, DNR status, power-of attorney (POA) status, and living will (LW) status. We generated descriptive statistics, and used logistic regression to evaluate predictors of DNR status.

**Results:**

A total of 754 patients over 20 months met inclusion criteria; 533 (70.7%) were white, 351 (46.6%) were male, and the median age was 66 years (IQR 54–78). DNR orders were found in 124 (16.4%, 95% CI [13.9–19.1%]) patients. In univariate analysis, there was a significant difference in DNR by gender (10.5% female vs. 6.0% male with DNR, p=0.013), race (13.4% white vs. 3.1% non-white with DNR, p=0.005), and age (4.0% <65 years; 2.9% 65–74 years, p=0.101; 3.3% 75–84 years, p=0.001; 6.2% >84 years, p<0.001). Using multivariate logistic regression, we found that factors associated with DNR status were gender (OR 1.477, p=0.358, note interaction term), POA status (OR 6.612, p<0.001), LW (18.032, p<0.001), age (65–74 years OR 1.261, p=0.478; 75–84 years OR 1.737, p=0.091, >84 years OR 5.258, P<0.001), with interactions between POA and gender (OR 0.294, P=0.016) and between POA and LW (OR 0.227, p<0.005). Secondary analysis demonstrated that DNR orders were not significantly associated with death during admission (p=0.084).

**Conclusion:**

Age, gender, POA, and LW use are predictors of ECF patient DNR use. Further, DNR presence is not a predictor of death in the hospital.

## INTRODUCTION

End-of-life medical care is responsible for a substantial portion of healthcare expenditures.^1^–^3^ Advance directives (AD) exist to convey a patient’s wishes regarding medical interventions when they no longer have capacity to express their wishes themselves. In the proper setting, they have the potential to prevent interventions not wanted by the patient at the end of life. In the emergency department (ED), the documented existence of a do-not-resuscitate (DNR) order may affect initial treatment decisions. Further, other forms of ADs, which include living wills and healthcare power of attorneys (POA), can inform the emergency physician with regards to patient preferences when the patient can no longer contribute to the decision-making process.

Patients requiring care in an extended care facility (ECF) are at high risk for both illnesses with high mortality potential as well as acute and chronic cognitive impairment.^4^–^6^ However, a lack of ECF communication regarding patient history^7^ and ADs^8^ has been demonstrated in patients transferred from ECFs to EDs. The most recent national survey of nursing homes demonstrated that 65.3% of residents had some kind of AD, of which 55.9% were do-not-resuscitate orders.^5^ Factors associated with DNR status in the nursing home have been described, including age, education, living children, length of stay, ambulatory status and ethnicity.^9^ However, despite the high prevalence of documentation of ADs in the ECF setting, there is a concern for the transfer and recognition of this documentation in the ED and inpatient setting, given the finding that 46.4% of transfers neglect to include information regarding ADs.^7^ Further, data support that ADs are often not recognized or are misinterpreted by physicians with regards to DNR status.^10^

We performed an epidemiologic analysis of the prevalence of documentation of ADs, including do-not-resuscitate orders, living wills and healthcare POAs, among residents of ECFs presenting to the ED. We focused on the presence of DNR orders, as these are most relevant to medical care in the ED and sought to describe the prevalence of DNR orders in patients sent to the ED from extended care facilities. Secondly, we sought to identify the relationship between age and DNR status while controlling for other variables. We felt this necessary, as age has been treated variably in previous studies. For example age has been broken into greater than and less than 65 or 75,^8^,^11^ or into decades or larger categories^9^,^12^–^14^ when relating it to DNR status in the nursing home. For this secondary analysis, we hypothesized that documentation of DNR status would become more prevalent with increasing age.

## METHODS

This study was conducted in the ED of a university-based, urban academic hospital in Ohio with an annual census of approximately 70,000 visits per year. We performed a retrospective, observational cohort study from our ED electronic medical record of patients originating from extended care facilities. This study was approved with a waiver of informed consent due to its retrospective nature by the Ohio State University Biomedical institutional review board. Eligible patients were identified via the “Source of Admission” data field as designated by the triage nurse upon arrival of the patient to the emergency department. Within our electronic medical record definitions, ECFs included skilled nursing facilities, long-term acute care facilities, assisted living facilities and rehabilitation facilities. For a consecutive 20-month period from October 20, 2007, to May 11, 2009, all patients designated as presenting from an ECF were identified and reviewed for inclusion in the study. We excluded return visits during the time period, as well as patients who were under the age of 18. Finally, during the data collection process the site of origin was verified via manual review of documentation of the site of origin and patients who were incorrectly coded during triage as originating from an ECF were removed from the final sample. We based our sample size calculation on the large range in prevalence of DNR orders reported in the literature and clinical experience, and we estimated 750 patients would provide an acceptable confidence interval of ±3.5% around the expected sample prevalence of DNR orders.^15^

We collected data from the ED electronic medical record (ED PulseCheck, Picis, Inc., Wakefield, MA) and the hospital electronic medical record (eResults, Lakeland HealthCare, St. Joseph, MI) via chart review. The complete electronic medical record for the identified visit and hospitalization (if applicable) as well as any information from the previous month were assessed. Variables that were collected included age, gender, race, mode of arrival, triage acuity level, disposition from the ED, in-hospital death, and advance directive status. We analyzed age both continuously and by decade. Although race was reported as White, Black or other in the medical record, we dichotomized race as White and non-White, as <1% of patients were reported as “other.” Mode of arrival was reported as via private vehicle, via private ambulance or wheelchair van, or via EMS. Acuity was reported using the Emergency Severity Index (ESI),^16^ which is a five-level scale that ranges from 1 – life threatening to 5 – non-urgent. At our institution the Emergency Severity Index initially is assigned at the time of triage, and then updated at the time of disposition. For the data analysis, we changed the initial and final ESI to a 1–4 scale with non-urgent (5) and semi-urgent combined (4), as only one patient was non-urgent. Disposition from the ED was reported as admission or discharge.

In Ohio, ADs consist of DNR-CC (comfort care only), DNR-CCA (full resuscitation up to the point of actual respiratory or cardiac arrest), living wills (LW) and healthcare POA. A fourth category was identified as advance directives not otherwise specified (AD-NOS) in the electronic medical record. In our institution, the documentation of a non-specified AD referred to a living will, so ADs not otherwise specified and living wills were combined for the statistical analysis. The presence of an advance directive could have been documented by physicians, nurses, or social workers.

The primary outcome was the presence of any form of DNR (DNR-CC, DNR-CCA, DNR not otherwise specified) documentation transferred with the patient from the ECF, as reported in the ED record or admission assessment. Secondary outcomes included death in hospital and disposition from the ED.

We generated descriptive statistics for all variables. Given the interest in age as related to other variables of interest, we used chi-square tests to compare age by decade to other study variables. When significant associations were noted among multilevel variables, we used adjusted standardized residuals to determine where the differences lay.

To identify independent predictors of DNR status, initial analysis was conducted using univariate logistic regression. In the logistic regression models, we examined the continuous variable age for linearity in the logit using a locally weighted smoothed scatter plot and fractional polynomial analysis.^17^ Age, if treated as a continuous variable, was found to be most accurately represented as a cubed variable. The use of age cubed in the models resulted in difficult to interpret odds ratios (e.g. 1.000003). Given the difficulty of interpretation of OR with age as a cubed variable, we divided age as a categorical variable at cut-points of <65, 65–74, 75–84, and >85 years. We used multivariate logistic regression to evaluate predictors of DNR status. An initial full model was built including all terms with a likelihood ratio with p<0.25 in the univariate analysis and biologic plausibility. We built a parsimonious model by removing terms in a backwards, manual, step-wise fashion, requiring a p<0.05 via the likelihood ratio test for retention. Interaction terms were tested for all remaining variables. We tested all models for goodness of fit using the Hosmer-Lemeshow goodness-of-fit test, and discrimination was tested using an ROC curve. We performed all statistical analyses using STATA/SE 10.1 (STATACorp, College Station, TX).

## RESULTS

We identified 1,736 patients documented as originating from an ECF over a consecutive 20-month time period. Of these initial patients, 211 were excluded by repeat visit, 36 were excluded because the patient was under the age of 18, and 735 were excluded because they were incorrectly coded with ECF as the source of admission. The remaining 754 patients constituted the study population, of which 351 were male (46.6%, 95% CI [49.8–57.0%]) and 533 were White (70.7%, 95% CI [67.4–73-9%]). The age range was 19 to 102 years old, with a median age of 66 (IQR 54–78). Additional descriptive characteristics of the patient population are listed in Table 1. There were a total of 124 DNR orders identified in this cohort (16.4%, 95% CI [13.9–19.1%]), of which 44 were DNR-CC (5.8%, 95% CI [4.2–7.5%]), 74 were DNR-CCA (9.8%, 95% CI [7.7–11.9%]), and six were DNR not-otherwise-specified (0.8%, 95% CI [0.2–1.4%]). There were 504 admissions from the ED (66%, 95% CI [63.5–70.2%]), and one death in the ED (0.13%, 95% CI [−0.12–0.39%]), with a total of 27 deaths during admission (3.7%, 95% CI [2.4–5.1%]). There were 14 missing initial triage levels found in the data set, which were replaced with the final ESI recorded. No other missing data were identified.

The univariate relationships with the primary outcome of presence of DNR are shown in Table 1. Age as a continuous variable was found to lack linearity in the logit. We therefore categorized age as <65, 65–74, 75–84, and ≥85. The last triage score prior to disposition was not included in the multivariate model, though it met criteria in the univariate analysis, because this status was generally assigned after DNR status for the patient became known and there was concern for substantial collinearity with initial triage level, as the urgency level infrequently changed through the ED stay.

We further investigated age with a univariate comparison of categorical age groups with other variables as noted in Table 2. The adjusted standardized residuals were calculated for all significant univariate associations between age and related variables. First triage score was significantly associated with age. The residuals from chi-squared analysis demonstrated that the >84 year old group had lower acuity, as did the <65 year old group. DNR orders, living wills and POAs were all significantly associated with age, with residuals indicating a significantly increased prevalence with increasing age. A POA was most prevalent in all age groups compared to other forms of ADs, and DNR orders were the least prevalent form of AD.

Results of the multivariate analysis for the outcome DNR status are reported in Table 3. The initial complete model included gender, race, power-of-attorney, living will, age by decade, and mode of arrival. In the final model after removal of non-significant variables, gender, age by decade, POA status, and living will status were found to be significant predictors of DNR status. The test for interactions in this final model demonstrated a significant interaction of gender with POA and POA with living will, indicating that males with a POA had a decreased association with DNR orders (interaction OR=0.29, p<0.016), as did patients with both a POA and a living will (interaction OR=0.23, p<0.005). The Hosmer-Lemeshow goodness-of-fit demonstrated no evidence of a lack of fit (p<0.614) and the area under the curve indicated good discrimination (0.846). We further clarified the effect of the interaction term on gender and DNR status in the final model. Compared to females, males with POAs were less likely to have documented DNR status, while males without POAs were more likely than females to have documented DNR status.

We also generated a third, simplified model to avoid possible interactions by including only gender, living will, and age. The removal of POA and its resulting interactions did not affect the odds ratios for age by decade. There was no significant difference between males and females regarding DNR status when the interaction term was removed in the simplified model. Age and living will remained significant predictors of DNR status.

The secondary analysis yielded associations regarding the outcomes of admission and death. We performed univariate and logistic regression using ED disposition and in-hospital death as outcomes. The analysis focusing on ED disposition demonstrated that presence of a POA (OR 2.713, p<0.001) and the presence of an AD (OR=2.86, p<0.001), after adjusting for initial triage score (Urgent OR=9.81, p<0.000; Emergent OR=19.85, p<0.000; Life Threat OR=39.07, p<0.000) was significantly associated with increased odds of admission from the ED. The analysis further demonstrated that the presence of a POA (OR 2.62, p=0.017), after adjustment for male sex (OR=3.00, p=0.009) was significantly associated with death during admission. The initial ED triage score was not associated with the presence of a living will (p=0.230) but was significantly associated with POA (p<0.002), with more than expected powers-of-attorney in the emergent triage group and less in the semi-urgent group. Although patients with DNR orders were more likely to be admitted to the hospital (OR 2.8, p<0.001), DNR orders were not significantly associated with death during admission (p=0.084). Further, there was no significant association between DNR-CC orders (p=0.063) or DNR-CCA orders (p=0.870) and in-hospital death when analyzed separately.

## DISCUSSION

Our study demonstrated an overall prevalence of DNR orders of 16.4% in the population presenting to the ED from ECFs overall. This number is in stark contrast to previous studies, which have demonstrated a high prevalence of DNR orders in patients transferred to the ED from skilled nursing facilities (32–64%),^5^,^8^,^9^,^11^,^12^,^18^,^19^ and from ECFs (68–77%)^20^. There are three potential external correctable sources for this discrepancy, which would include a deficit in the existence of the documentation in the ECF population seen in the ED, a lack of transfer of the paperwork from facilities,^7^ or a lack of documentation of these orders in the medical chart in the ED. Alternatively, given the increasing number of hospices providing care in nursing homes and the increased utilization^21^ of hospice in general, it is possible that patients with DNR orders are less frequently being sent to the ED and thus not represented in this study. Regardless, given the paucity of research examining the larger ECF population and specific factors associated with DNR orders, this population demonstrates many potential avenues for further investigation and areas of improvement in advance care planning.

Although significant in the univariate analysis, race was not significant in the multivariate analysis when controlling for age, mode of arrival, POA, and living will. Some authors have shown a relationship with race, which was not found in this study, possibly due to the inclusion of confounders in past analyses.^8^,^9^,^11^,^12^

In the multivariate analysis, patients of increasing age were significantly more likely to present with a DNR order. Previous studies have consistently demonstrated similar associations with age.^8^,^9^,^14^,^22^ Age was broken out further in Table 2, and demonstrated significant associations with the presence of DNR orders, living wills, and healthcare POAs. While the oldest of the geriatric population has a high prevalence of POA (59.5%), living will (40.5%) and DNR orders (40.5%), the younger geriatric population has a significantly lower prevalence of these documents. This discrepancy, which is consistent with previous literature, demonstrates a potential for discussions in the younger geriatric ECF population regarding advance care planning, as well as instituting policies for the transfer of these documents with these patients to the ED.

The relationship between gender and DNR status in the multivariate models is less clear. Previous studies have shown either a trend towards increased DNR prevalence in women or a lack of association of gender with DNR orders.^8^,^9^,^11^,^12^ Gender was not significant in the third simplified model. Gender was significant in the model, which included POA and living will as independent variables, but was involved in an interaction with POA. When considering this interaction, men with a POA were less likely than women to have DNR status, but men without a POA were more likely than women to have DNR status. This may indicate that, among males, discussions of medical decision-making are excluding discussions of DNR orders. Alternatively, it may be that DNR orders are felt unnecessary in cases where a POA has been appointed. This trend has not been reflected in the literature previously, and indicates that additional investigation is warranted to understand the nuances of this association.

The interaction between living wills and powers-of-attorney demonstrates a decreased likelihood of the presence of a DNR if both documents are in place. While this interaction could be an artifact of the high prevalence of POAs and living wills, it may indicate that patients who are able to have end-of-life discussions and sign these documents are not being approached regarding DNR orders or are not willing to enact DNR orders. Additionally, suggested by the clinical experience of the authors, another possibility is that many patients and their families assume that a living will and POA encompass DNR status. The trend of age remained consistent throughout the model, regardless of the inclusion of the interaction terms.

We examined admission rates and death in the context of the prevalence of DNR orders, as well as patient population characteristics. Although age was not significantly associated with admission, DNR status was associated with an increased odds of admission. Interestingly, POAs and living wills were significantly associated with admission, potentially demonstrating an artificial increase, as social workers at our institution are tasked with obtaining these documents from the family and ECF upon admission. An alternative explanation could be that patients with these documents are more moribund; however, when looking at the initial triage score, this trend only held true for POAs. Finally, only male gender and POA were significantly associated with death during admission.

The literature has not addressed the hazard of death in patients with DNR orders in general. Only a few studies have approached the relationship, specifically noting increased post-operative mortality in patients with pre-operative DNR orders^23^ and a high prevalence of DNR orders in patients who expired in the nursing home setting.^6^ However, in our secondary analysis DNR status was not associated with death in the hospital. Further, DNR orders were not associated with higher initial triage severity indexes, demonstrating that patients who presented with DNR orders were not considered more or less acute upon arrival to the ED and were not more likely to expire during admission than those without orders. These trends are counterintuitive given the association between advanced illness and DNR orders.^22^ Patients with DNR orders were almost three times as likely to be admitted than those without DNR orders, a trend that was independent of age in multivariate analysis. Further research is needed to explore the causal relationship between DNR orders, admission and survival to discharge.

## LIMITATIONS

This study is a retrospective chart review, which does preclude several inherent and modifiable limitations.^24^ To minimize error, only one abstractor of data from the medical record was used, with a supervising principal investigator to review discrepancies within the medical record. However, this abstractor was not blinded to the purpose of the study. The data were collected using a standardized list of variables, and all chart documentation from the visit and the previous month was reviewed for every patient. As we selected cases based on a source of origin code recorded by the triage nurse, and almost half of the charts were excluded by incorrect coding, there is a large potential for patient selection error. In retrospect, there was a pattern noted among specific triage nurses who coded all ambulance runs as originating from an ECF, which accounted for the majority of the miscoded patients. However, bias could exist due to missing patients from ECFs who were not identified via miscoding and thus not included in the study. The only missing data element within the collected data set was the 14 missing initial triage levels, which were directly imputed from the final triage levels noted at the point of disposition from the ED. With regards to in hospital death as a secondary outcome, this does not capture those patients who were discharged home on hospice to avoid in-hospital death; thus, the number of association of death and DNR orders may be underrepresented in this study.

## CONCLUSION

There exists a large body of data regarding DNR orders in nursing homes; however, the transfer of these orders to the ED from extended care facilities is less well understood. Given the potential gaps in transferred data and the critical nature of these documents in patient care, it is unsettling that the prevalence of these orders in patients transferred to the ED at the time of assessment is so low. Given the trend towards advance directives and DNR orders in the older geriatric population, there exist potential windows of opportunity to discuss patients’ wishes to establish advance directives at an earlier age, as well as to study further the relationship between gender, race and DNR status. Finally, the associations between DNR orders, admission and in-hospital death warrant further investigation.

## Figures and Tables

**Table 1:** Characteristics of the population with univariate analysis for odds of do not resuscitate in patients presenting to the emergency department from extended care facilities.

	Total n (%)	With DNR (%)	Without DNR (%)	OR (5% CI)	P-value
Gender					
Male	351 (46.6)	45 (6.0)	306 (40.6)	0.60 (0.41–0.90)	0.013
Age by decade					
<65	346 (45.9)	30 (4.0)	316 (41.9)	reference	
65–74	164 (21.8)	22 (2.9)	142 (18.8)	1.63 (0.91–2.93)	0.101
75–84	128 (17.0)	25 (3.3)	103 (13.7)	2.56 (1.44–4.55)	0.001
>84	116 (15.4)	47 (6.2)	69 (9.2)	7.18 (4.24–12.15)	<0.001
Race					
White	533 (70.7)	101 (13.4)	432 (57.3)	2.01 (1.24–3.26)	0.005
Transportation					
Private vehicle	30 (4.0)	7 (0.9)	23 (3.1)	reference	
Ambulance	428 (56.8)	52 (6.9)	376 (49.9)	0.45 (0.19–1.11)	0.084
EMS	296 (39.3)	65 (8.6)	231 (30.6)	0.92 (0.38–2.23)	0.863
Initial urgency (ESI)					
Life threat	12 (1.6)	1 (0.1)	11 (1.5)	0.82 (0.08–8.75)	0.868
Emergent	277 (36.7)	53 (7.0)	224 (29.7)	2.13 (0.62–7.28)	0.228
Urgent	435 (57.7)	67 (8.9)	368 (48.8)	1.64 (0.48–5.56)	0.428
Semi-urgent	29 (3.8)	3 (0.4)	26 (3.4)	combined reference	
Non-urgent	1 (0.1)	0 (0.0)	1 (0.1)	combined reference	
Final urgency (ESI)					
Life threat	19 (2.5)	1 (0.1)	18 (2.4)	1.28 (0.08–21.86)	0.866
Emergent	300 (39.8)	66 (8.8)	234 (31.0)	6.49 (0.86–48.94)	0.070
Urgent	411 (54.5)	56 (7.4)	355 (47.1)	3.63 (0.48–27.40)	0.212
Semi-urgent	23 (3.1)	1 (0.1)	22 (2.9)	combined reference	
Non-urgent	1 (0.1)	0 (0.0)	1 (0.1)	combined reference	
Advance directive (AD)					
Power of attorney	317 (42.0)	94 (12.5)	223 (29.6)	5.72 (3.68–9.90)	<0.001
Living will (LW)	89 (11.8)	37 (4.9)	52 (6.9)	9.93 (6.46–15.25)	<0.001
Other AD	189 (25.1)	83 (11.0)	106 (14.1)	combined with LW	
Disposition					
Admit	504 (66.8)	103 (13.7)	401 (53.2)	2.80 (1.70–4.60)	<0.001

*DNR,* do not resuscitate; *EMS,* emergency medical services*; ESI;* emergency severity index; *OR,* odds ratio

**Table 2:** Univariate analysis of population characteristics based on age.

	<65 (%)	65–74 (%)	75–84 (%)	>84 (%)	P-value (chi)
Gender					
Female	173 (50.0)	87 (53.1)	69 (53.9)	74 (63.8)	
Male	173 (50.0)	77 (47.0)	59 (46.1)	42 (36.2)	0.083
Race					
White	235 (67.9)	119 (72.5)	93 (72.7)	86 (25.9)	
Other	111 (32.0)	45 (27.4)	35 (27.3)	30 (74.1)	0.482
Transportation					
Private vehicle	13 (4.3)	3 (1.8)	6 (4.7)	6 (5.2)	
Ambulance or wheelchair	200 (57.8)	97 (59.2)	70 (54.7)	61 (52.6)	
EMS	131 (37.9)	64 (27.8)	52 (40.6)	49 (42.2)	0.707
Initial Urgency (ESI)					
Life Threat	5 (1.5)	1 (0.6)	6 (4.7)	0 (0.0)	
Emergent	120 (34.7)	69 (42.1)	49 (38.3)	39 (33.6)	
Urgent	208 (60.1)	84 (51.2)	70 (54.7)	73 (62.9)	
Semi/non-urgent	13 (3.8)	10 (6.1)	3 (2.3)	4 (3.5)	0.038
Final urgency (ESI)					
Life threat	8 (2.3)	5 (3.1)	6 (4.7)	0 (0.0)	
Emergent	133 (38.4)	62 (37.8)	59 (46.1)	46 (39.7)	
Urgent	195 (56.4)	89 (54.3)	60 (46.9)	67 (57.8)	
Semi/non-urgent	10 (2.9)	8 (4.9)	3 (2.3)	3 (2.6)	0.279
Advance directive					
Power of attorney	111 (32.1)	70 (42.7)	67 (52.3)	69 (59.5)	<0.001
Living will	60 (17.3)	43 (26.2)	44 (34.4)	47 (40.5)	<0.001
DNR	30 (8.7)	22 (13.4)	25 (19.5)	47 (40.5)	<0.001
Disposition					
Admit	224 (64.7)	107 (65.2)	90 (70.3)	83 (71.6)	
Discharge	122 (35.3)	57 (34.8)	38 (26.7)	33 (33.0)	0.434

*DNR,* do not resuscitate; *ESI;* emergency severity index

**Table 3:** Multivariate logistic regression of population based on do-not-resuscitate status.

	Complete model**		Final model†		Simplified model‡	
	
	OR (95% CI)	P-value	OR (95% CI)	P-value	OR (95% CI)	P-value
Male	0.69 (0.41–1.05)	0.078	1.48 (0.64–3.39)	0.358	0.65 (0.41–1.02)	0.063
White	1.55 (0.89–2.69)	0.120				
POA	2.01 (1.18–3.44)	0.010	6.61 (2.89–12.12)	<0.001		
Living will (LW)	6.32 (3.81–10.50)	<0.001	18.03 (7.50–43.35)	<0.001	9.03 (5.75–14.17)	<0.001
65–74	1.24 (0.65–2.36)	0.513	1.26 (0.66–2.39)	0.478	1.31 (0.70–2.46)	0.402
75–84	1.57 (0.83–2.99)	0.168	1.74 (0.92–3.30)	0.091	1.76 (0.94–3.30)	0.079
>84	4.47 (2.62–8.67)	<0.001	5.26 (2.87–9.64)	<0.001	5.38 (2.99–9.66)	<0.001
Ambulance	0.56 (0.18–1.75)	0.318				
EMS	1.02 (0.33–3.17)	0.977				
POA × male			0.29 (0.11–0.80)	0.016		
POA × LW			0.23 (0.08–0.64)	0.005		

*POA,* power of attorney; *EMS,* emergency medical services*; OR,* odds ratio*; CI,* confidence interval

** Goodness of fit (p<0.931), discrimination=0.84.

† Goodness of fit (p<0.614), discrimination=0.85.

‡ Goodness of fit (p<0.521), discrimination=0.82.
